# The Role of Buckling in the Estimation of Compressive Strength of Corrugated Cardboard Boxes

**DOI:** 10.3390/ma13204578

**Published:** 2020-10-14

**Authors:** Tomasz Garbowski, Tomasz Gajewski, Jakub Krzysztof Grabski

**Affiliations:** 1 Institute of Structural Analysis, Poznan University of Technology, Piotrowo Street 5, 60-965 Poznań, Poland; tomasz.garbowski@put.poznan.pl (T.G.); tomasz.gajewski@put.poznan.pl (T.G.); 2 Institute of Applied Mechanics, Poznan University of Technology, Jana Pawła II Street 24, 60-965 Poznań, Poland

**Keywords:** corrugated board, McKee formula, buckling, orthotropic panels, laboratory tests, box strength

## Abstract

This paper presents analytical methods for estimating the static top-to-bottom compressive strength of simple corrugated packaging, in which the torsional and shear stiffness of corrugated cardboard as well as the panel depth-to-width ratio are included. The methods are compared herein with a basic and more detailed buckling description with the successful McKee formula, which is over fifty years old but still widely used among packaging designers and quality control departments. Additionally, the assumptions and applied simplifications used in the literature are analyzed, and the limits of applicability of different versions of the selected methods are checked. Finally, all approaches are verified with the experiment results of various packaging designs made of corrugated cardboard. The results show that, for certain proportions of dimensions of simple flap boxes, simplified methods give an even two times larger estimation error than the analytical approach proposed in the paper. Furthermore, it is evidenced that including all flexural, torsional and shear stiffnesses in the buckling force estimation gives a very precise prediction of the box compressive strength for the full range of package dimensions.

## 1. Introduction

Corrugated cardboard has been gaining popularity in recent years and is becoming one of the leading materials in the packaging industry. However, this was not always the case; only a dozen years ago, cardboard packaging was associated mainly with disposal. Internet sales were at a much lower level compared to what we observe today. In addition, most companies and consumers did not attach much importance to environmental protection, and existing regulations did not require the monitoring of the packaging industry, which led to the expansion of another material—plastic. Fortunately, companies quickly realized that plastic not only harms the environment but also impairs their brands’ reputations. Additionally, consumers around the world have increased their demands for environmental responsibility, which has forced many companies to change. One of the best alternatives became recyclable corrugated cardboard, mainly due to its biodegradable nature.

Today’s corrugated cardboard packaging no longer consists of boring, gray-brown transport boxes. Packaging may be produced with multicolored prints and in various shapes. This makes it very attractive for e-commerce companies and their customers. It often happens that packaging must meet both aesthetic and strength criteria. The best example is the so-called shelf-ready packaging (SRP) or retail-ready packaging (RRP)—branded boxes that are ready for retail display. To optimally choose the quality of corrugated cardboard for this type of packaging, numerical models [1,2,3,4] based on homogenization methods [5,6,7,8,9,10,11] are necessary. Models based on analytical equations or empirical formulas are sufficient only for simple flap constructions (see [12,13,14,15]).

One of the most popular methods of estimating the strength of cuboid-shaped packaging is the approach proposed in the 1960s by McKee et al. [12]. This method uses the basic parameters of corrugated cardboard and empirically determined correction factors that increase the accuracy of the results obtained. Unfortunately, the introduction of correction factors reduces the universality of the method, mainly because their values have to be determined individually for practically every cardboard quality and every packaging design. To better understand the limitations of this method, commonly known as the McKee formula, it is necessary to study the performance of simplified methods for a box strength estimation, comparing them to other, more sophisticated approaches, and to confront all methods with real experimental data, which will be shown later in this work.

The prediction of corrugated cardboard boxes’ strength based on relatively simple formulas has been of interest to numerous groups of scientists for many years. In their research, they used various approaches and considered different phenomena and properties of paper, cardboard and boxes. In 1952, Kellicutt and Landt [16] proposed a formula for the compressive strength of corrugated boxes based on the box compression test (BCT). In this formula, they took into account the overall ring crush strength of linerboards, box perimeter, flute and box constants. A relationship between the critical force in the BCT and the dimensions of the box has been established by Maltenfort and described in the paper [17] from 1956. His formula is based on the Concora liner test (CLT), an empirical constant related to the board and dimensions of the box. The widely used formula of McKee, Gander and Wachuta [12] related the compression strength to the edge crush test (ECT), flexural stiffness of the board and box dimensions. In the formula proposed by Whitsitt et al. [18], a time to failure was estimated based on the ECT, flexural stiffness of the board, perimeter of the box, buckling ratio, stacking time and applied load ratio. Modified McKee formulas for C-flute corrugated fiberboards were proposed by Allerby et al. [19], who changed the constants and exponents of the McKee formula. Similarly, in 1987, Schrampfer et al. proposed a modified McKee relationship for a wide range of cutting methods and equipment [20]. In 1989, Kawanishi in [21] proposed a formula for the compression strength of boxes, based on some parameters relevant from a practical point of view. The formula included the weight of linerboards and corrugated fiberboards, take-up factor, average corrugation count, thickness of the fiberboard, box perimeter, box type, printed ratio and sidewall moisture content. Batelka et al. [22] expanded the applicability of the McKee formula to a wider range of containers. They also considered the influence of the width and depth of the box. In the work of Urbanik et al. [23], the formula for the compression strength of boxes was based inter alia on Poisson’s ratio. The finite element method (FEM) was used to calculate the compression strength. A similar approach to the analysis of box strength using FEM computations has been seen in the literature for many years. Nordstrand and Carlsson compared the effective transverse shear moduli of corrugated boards obtained from FEM simulations with experimental results and analytical predictions [24,25]. Urbanik and Saliklis applied FEM to observe the buckling phenomena in the corrugated boxes [26]. A valuable review of the analytical approach and numerical prediction of box strength can be found in [27].

All the above-mentioned analytical and numerical methods for estimating the compressive strength of corrugated cardboard packaging require the determination of a specific set of material parameters. Even if the key to choosing a method for further analysis is the ease of calibrating the computational models, we cannot avoid laboratory work to obtain the necessary parameters. In the simplest models, it is enough to estimate the edge-loaded compressive strength of the corrugated cardboard and its thickness; for more demanding models, the bending stiffness of the corrugated cardboard in the machine and cross direction is also required. Unfortunately, laboratory testing of the torsional and shear stiffness of corrugated cardboard is seldom performed. Therefore, even these days, equations similar to those proposed by McKee et al. are still seen as the simplest and also the most accurate predictors of the load capacity of regular flap boxes. The success of these methods is based on the use of those cardboard parameters that are easily obtained in the laboratory and on empirical coefficients that make it possible to accurately match the results to the experimental data. However, if the transverse and bending stiffnesses are included in the model, first, more accurate results can be obtained for variable geometries of the boxes, and second, problematic correction factors can be ignored. Fortunately, more and more industrial laboratories have been recently equipped with advanced devices for measuring all the material parameters of corrugated cardboard. Therefore, the use of tools that use advanced mechanical methods to estimate the load capacity of corrugated cardboard packaging is gaining popularity.

## 2. Materials and Methods

### 2.1. Ultimate Compressive Strength of a Plate

**Figure 1:**
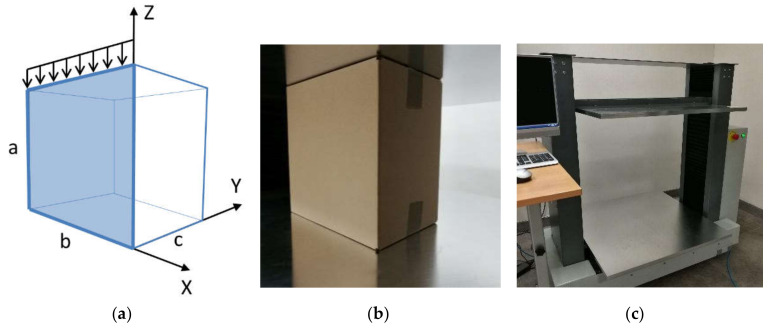
(**a**) A single panel of width b and height a separated from cardboard packaging as a supported plate under compression; (**b**) cardboard packaging in press during box compression testing; (**c**) box compression press used in laboratory tests [29].

As was mentioned already, the most popular yet the simplest approach was proposed by McKee et al. [12], who began developing the formula by determining the ultimate plate load $P_{f}$ for a rectangular panel of dimensions a×b, loaded vertically on the upper edge. The analyzed plate is a separated panel of a box (Figure 1a), despite the fact that during the compression test the box is loaded as a three-dimensional structure (Figure 1b,c). All four edges of the plate are pinned in the Y direction (out-of-plane), and the bottom edge is additionally supported along b in the Z direction. The basis for the derivation is the empirical formula [28] for the load $P_{f}$ at failure [N/mm], which in accordance with the first authors’ assumption is the result of a combination of compressive strength and buckling force, namely:

(1) $$\frac{P_{f}}{P_{cr}}=k{\left(\frac{ECT}{P_{cr}}\right)}^{r},$$

where k is a constant, r is an exponent, $r\in \;\left(0,1\right)$ (most often r=3/4), ECT is the edge-loaded compressive strength of corrugated cardboard, in N/mm, and $P_{cr}$ is a critical load, in N/mm, resulting from the buckling phenomenon of the vertical walls of the box.

This formula was later modified by Urbanik et al. [23,26] to take into account the case in which elastic buckling does not happen and the nonlinear material effect plays a key role:

(2) $$\frac{P_{f}}{ECT}=k{\left(\frac{P_{cr}}{ECT}\right)}^{un},$$

where n=1−r. Input u=1 corresponds to elastic buckling when $ECT>P_{cr}$, and u=0 corresponds to inelastic buckling when the ultimate load $P_{f}$ depends only on ECT, namely:

(3) $$P_{f}=k\;ECT.$$

In both cases, the critical load for a rectangular orthotropic plate (a selected panel of a corrugated cardboard box, see Figure 1a) has the following form [30,31,32]:

(4) $$P_{cr}^{b}=\frac{\pi^{2}\;\sqrt{D_{11}D_{22}}\;}{b^{2}}k_{cr},$$

where $P_{cr}^{b}$ is the critical load of the panel of dimensions a×b (a—panel height, b—panel width), $D_{11}$ is the bending stiffness in the machine direction (MD), in Nmm, $D_{22}$ is the bending stiffness in the transverse direction (CD), in Nmm, b is the plate width [mm], and $k_{cr}$ is a dimensionless buckling coefficient that depends, among others, on the ratio a/b, boundary conditions applied to the plate edges, the buckling mode and a material characteristic.

### 2.2. Buckling of Rectangular Orthotropic Panel

Corrugated cardboard, like other fibrous materials, is characterized by orthotropy—its mechanical properties change depending on the direction (CD/MD). The buckling coefficient in such a case has the form:

(5) $$k_{cr}=\sqrt{\frac{D_{11}}{D_{22}}}{\left(\frac{mb}{a}\right)}^{2}+2\frac{\left(D_{12}+2D_{66}\right)}{\sqrt{D_{11}D_{22}}}+\sqrt{\frac{D_{22}}{D_{11}}}{\left(\frac{a}{mb}\right)}^{2}.$$

By substituting Equation (5) into Equation (4), we get the critical load of an orthotropic plate of any dimensions a and b, which now has the following definition:

(6) $$P_{cr}^{b}=\frac{1}{\alpha^{2}}\left(D_{11}\alpha^{4}+2\left(D_{12}+2D_{66}\right)\alpha^{2}\beta^{2}+D_{22}\beta^{4}\right),$$

where:

(7) $$\alpha =\frac{m\pi }{a},\;\beta =\frac{\pi }{b},$$

(8) $$D_{11}=\frac{1}{w}{\underline{E}}_{11}I,\;D_{22}=\frac{1}{w}{\underline{E}}_{22}I,$$

(9) $$D_{12}=\frac{{\underline{\nu }}_{21}}{w}{\underline{E}}_{11}I=\frac{{\underline{\nu }}_{12}}{w}{\underline{E}}_{22}I,\;D_{66}={\underline{G}}_{12}I,$$

(10) $$I=\frac{{\underline{h}}^{3}}{12},\;w=\left(1-{\underline{\nu }}_{12}{\underline{\nu }}_{21}\right),$$

where $\underline{h}$ is the effective cardboard thickness, ${\underline{E}}_{11}$ is the effective modulus of elasticity in the MD, ${\underline{E}}_{22}$ is the effective modulus of elasticity in the CD, ${\underline{G}}_{12}$ is the effective in-plane shear modulus, and ${\underline{\nu }}_{12}$ and ${\underline{\nu }}_{21}$ are effective Poisson’s coefficients in the plane. In the context of the modulus of elasticity, effective means homogenized or substituted; more details on this topic can be found in [5,6,7,8,9,10,11,33].

### 2.3. Buckling Including Transverse Shear Stiffness

If we also want to consider the transverse shear stiffness [34,35], ${\underline{G}}_{13}$ and ${\underline{G}}_{23}$, in order to obtain a more precise prediction, the formula for the critical load should take the following form [32]:

(11) $$P_{cr}^{b}=\frac{1}{\alpha^{2}}\frac{M}{N},$$

where:

(12) $$M=D_{11}\alpha^{4}+2\left(D_{12}+2D_{66}\right)\alpha^{2}\beta^{2}+D_{22}\beta^{4}+\left(\frac{\alpha^{2}}{A_{44}}+\frac{\beta^{2}}{A_{55}}\right)c_{1},$$

(13) $$N=1+\frac{c_{1}}{A_{44}A_{55}}+\frac{c_{2}}{A_{55}}+\frac{c_{3}}{A_{44}},$$

(14) $$A_{44}=\frac{5}{6}{\underline{G}}_{13}\underline{h},\;A_{55}=\frac{5}{6}{\underline{G}}_{23}\underline{h},$$

(15) $$c_{1}=c_{2}c_{3}-c_{4}^{2}>0,$$

(16) $$c_{2}=D_{11}\alpha^{2}+D_{66}\beta^{2}$$

(17) $$c_{3}=D_{66}\alpha^{2}+D_{22}\beta^{2}$$

(18) $$c_{4}=\left(D_{12}+D_{66}\right)\alpha \beta$$

This approach is crucial in cases where the corrugated cardboard is thicker (especially with a flute composition of B, C, BC or EB) and its transverse shear modulus is relatively low (e.g., due to unintentional crushing during printing).

### 2.4. Buckling in McKee’s Formula

The goal of the authors of the McKee formula was to maximally simplify the complicated equations describing the critical load of compressed orthotropic plates so that the use of these equations could be common and practical. The next step taken by the authors of the McKee formula was to simplify Equation (5) describing the constant $k_{cr}$ through the formula:

(19) $$k_{cr}={\left(\frac{mb}{\theta a}\right)}^{2}+{\left(\frac{\theta a}{mb}\right)}^{2}+2K,$$

where: $\theta =\sqrt{D_{11}^{-1}D_{22}}$ and K is a plate parameter assumed to be equal to 0.5, which means that $D_{12}+2D_{33}=0.5\sqrt{D_{11}D_{22}}$. It was also assumed that the plate has equal sides, i.e., b=c and therefore b=Z/4 (where Z is the box perimeter), which leads to further simplification of Equation (4) to the form:

(20) $$P_{cr}^{b}=\frac{{\left(4\pi \right)}^{2}\sqrt{D_{11}D_{22}}}{Z^{2}}k_{cr}.$$

### 2.5. Buckling of Square Isotropic Panel

In this section, for comparison we present the simpler formulas, in which the torsional and shear stiffnesses and/or panel depth-to-width ratios are neglected. For the isotropic material definition, we obtain:

(21) $$\frac{D_{12}+2D_{33}}{\sqrt{D_{11}D_{22}}}≅1.$$

As a result, the buckling coefficient defined in Equation (5) is reduced to:

(22) $$k_{cr}={\left(\frac{mb}{a}+\frac{a}{mb}\right)}^{2}.$$

Thus, the critical force can be described by the following equation:

(23) $$P_{cr}^{b}=\frac{\pi^{2}\sqrt{D_{11}D_{22}}}{b^{2}}{\left(\frac{mb}{a}+\frac{a}{mb}\right)}^{2}.$$

If we additionally assume (similarly as McKee) that the plate is a square (a=b, so m=1), the buckling coefficient simplifies to:

(24) $$k_{cr}=4,$$

and the buckling force reads:

(25) $$P_{cr}^{b}=\;\frac{4\pi^{2}\sqrt{D_{11}D_{22}}}{b^{2}}.$$

In the case of square orthotropic panels, Equation (5) simplifies to:

(26) $$k_{cr}=\frac{D_{11}+2\left(D_{12}+2D_{66}\right)+D_{22}}{\sqrt{D_{11}D_{22}}},$$

and the critical force reads:

(27) $$P_{cr}^{b}=\;\frac{\pi^{2}}{b^{2}}\left[D_{11}+2\left(D_{12}+2D_{66}\right)+D_{22}\right].$$

### 2.6. Determination of Box Compression Strength

In order to calculate the ultimate load capacity of a single panel of a box (Figure 1a), we need to multiply the value of force $P_{f}$ described by formula (1) by the i-th panel width b or c. To obtain the failure load for a whole box, we need to make a summation over all panels. Thus, in a general case, we get the following:

(28) $$BCT=2k\;ECT^{r}\left[\gamma_{b}{\left(P_{cr}^{b}\right)}^{1-r}b+\gamma_{c}{\left(P_{cr}^{c}\right)}^{1-r}c\right],$$

where $P_{cr}^{b}$ and $P_{cr}^{c}$ are the critical forces of panels of width b and c, respectively (see Figure 1a); $\gamma_{b}$ and $\gamma_{c}$ are the reduction coefficients, defined as:

(29) $$\gamma_{b}=\sqrt{\frac{b}{c}},\;\gamma_{c}=1\;if\;b\leq c,\;\gamma_{c}=\sqrt{\frac{c}{b}},\;\gamma_{b}=1\;if\;b>c.\;$$

### 2.7. Box Compression Strength—Simplification in McKee’s Formula

Substituting the buckling force derived from Equation (20) into Equation (1), we obtain:

(30) $$P_{f}=k\;ECT^{r}{\left(\sqrt{D_{11}D_{22}}\right)}^{1-r}Z^{-2\left(1-r\right)}{\left(4\pi \right)}^{2\left(1-r\right)}k_{cr}^{1-r},$$

which simplifies to:

(31) $$P_{f}=\hat{k}\;ECT^{r}{\left(\sqrt{D_{11}D_{22}}\right)}^{1-r}Z^{-2\left(1-r\right)},$$

where $\hat{k}$ (under the first authors’ assumption that $k_{cr}^{1-r}$ is constant if a/Z>1/7 and equals approximately 1.33, while r=0.746 and k=0.4215) is equal to:

(32) $$\hat{k}=1.33\;k\;{\left(4\pi \right)}^{2\left(1-r\right)}≅2.028.$$

To obtain the failure load for the whole box, it is sufficient to multiply Equation (31) by box perimeter Z. This is because the original assumption was that the width and length of a box are equal (i.e., b=c) so that there is no need to distinguish between different panels’ widths in the box. Thus, the ultimate compressive strength, also known as the long McKee formula, reads:

(33) $$BCT_{MK1}=\hat{k}\;ECT^{r}{\left(\sqrt{D_{11}D_{22}}\right)}^{1-r}Z^{2r-1}.$$

A further simplification, due to the authors’ empirical observations that $\sqrt{D_{11}D_{22}}≅66.1\;ECT\;h^{2}$, leads to the most known short form of McKee’s equation:

(34) $$BCT_{MK2}=\overset{ˇ}{k}\;ECT\;h^{2\left(1-r\right)}Z^{2r-1},$$

where $\overset{ˇ}{k}$, for a previously assumed r=0.746, equals:

(35) $$\overset{ˇ}{k}=\hat{k}\;{\left(66.1\right)}^{1-r}≅5.874,$$

So, finally, Equation (35) takes the form:

(36) $$BCT_{MK2}=5.874\;ECT\;h^{0.508}Z^{0.492}.$$

### 2.8. Practical Considerations

To compare the accuracy of Equation (28), which uses different definitions of the critical force (i.e., Equations (6), (11), (23), (25) and (27)) and both of the McKee formulas (Equations (33) and (35)), we have to have constant values of k and r in Equation (28), as suggested by other authors [12,13,14,15]. Consequently, let k=0.5 and r=0.75. The adoption of such values has some empirical basis, but unfortunately there is no mathematical or physical basis; in practice, any values can be adopted. This means that the value of ECT in Equation (28) tends to a higher exponent value, which may have some justification for short and rigid packaging, where the buckling of individual panels actually has less effect on the load capacity of the packaging. However, for tall packaging or packaging made of low-profile cardboard (E or F flutes), the tendency should be exactly the opposite.

The compressive strength of rectangular packages (with base dimensions b×c, as shown in Figure 1a) with the assumed typical values of k and r in Equation (28) reads:

(37) $$BCT=ECT^{0.75}\left[\gamma_{b}{\left(P_{cr}^{b}\right)}^{0.25}b+\gamma_{c}{\left(P_{cr}^{c}\right)}^{0.25}c\right],$$

where $P_{cr}^{b}$ and $P_{cr}^{c}$ are defined in:

(a) Equation (6)—for orthotropic rectangular plates,
(b) Equation (11)—for orthotropic rectangular plates including transversal shearing,
(c) Equation (23)—for quasi-isotropic rectangular plates (neglecting the torsional and shear stiffness of corrugated board),
(d) Equation (25)—for quasi-isotropic square plates (neglecting both the torsional and shear stiffness, and depth-to-width ratio of the corrugated board panel),
(e) Equation (27)—for orthotropic square plates (neglecting the panel depth-to-width ratio).

In all of the above equations describing the critical force $P_{cr}^{c}$, all definitions of width b should be replaced by c, e.g., Equation (23) should read:

(38) $$P_{cr}^{c}=\frac{\pi^{2}\sqrt{D_{11}D_{22}}}{c^{2}}{\left(\frac{mc}{a}+\frac{a}{mc}\right)}^{2}.$$

### 2.9. Box Strength—Summary

In determining box strength using the analytical approach presented in Section 2, the buckling force is a crucial factor. The approaches discussed in Section 2 are summarized in Table 1. The table shows which factors are taken into account in each approach. The most detailed modelling is represented by case B, while the simplest is represented by case D and case G (short McKee formula).

## 3. Results

### 3.1. Box Compression Strength—Experiment vs. Estimation

In the following section, experimental and computational examples regarding boxes are presented in order to show the applicability of selected analytical approaches. In the paper, the analyzed cases represent the simplest flap boxes, where McKee’s formula should have its lowest error.

**Figure 2:**
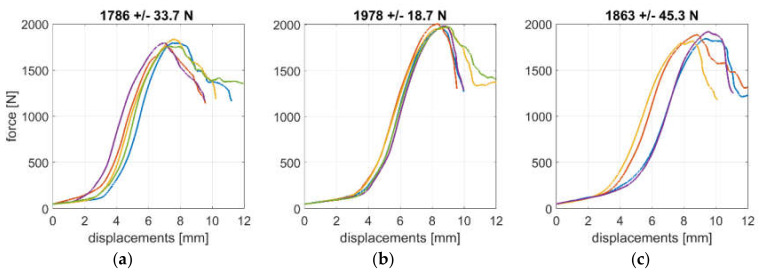
Example measurements from a box compression test machine [29] for different boxes: (**a**) 150 × 350 × 150 mm, (**b**) 200 × 300 × 250 mm and (**c**) 200 × 300 × 450 mm.

For the purposes of this study, the popular simple flap boxes with different aspect ratios were tested in a box compression testing machine, namely BCT-19T10 from FEMat [29]; see Figure 1c. According to the specifications, the machine measures the force up to 10 kN with a resolution of 0.1 N. The displacement accuracy is 0.001 mm. Four in-plane box sizes were considered, i.e., 250×250 mm, 300×200 mm, 350×150 mm and 400×100 mm. Each size was tested for three different heights: 150 mm, 250 mm and 450 mm. In order to get reliable results, 4–6 samples of each packaging were tested. The boxes were manufactured from three-layer 400 g/m^2^ corrugated cardboard (B flute) with a thickness equal to 2.85 mm, described here as 3B400 cardboard quality. The boxes were folded manually by one operator, and the flaps were taped at the top and bottom of the packaging. Before the test, each packaging was inspected for visual damage of the cardboard. A displacement control protocol was applied during the tests. Samples were laboratory conditioned according to TAPPI standard T402, i.e., temperature 23 °C ± 1 °C and relative humidity 50% ± 2%. Figure 2 presents examples of laboratory measurements for three selected designs of boxes, namely, 150×350×150 mm, 200×300×250 mm and 200×300×450 mm (width × length × height). The plots present the force versus displacement; a good repeatability of the measurement results is observed. The boxes were not inspected after the tests in order to determine the deformation and damage mechanism.

Laboratory tests and postprocessing computations according to Equations (8)–(10) allowed us to obtain the effective stiffnesses of the corrugated cardboard. The data for 3B400 cardboard quality used in the study are presented in Table 2. They were determined experimentally by using the effective homogenized approach embedded in the FEM in the laboratory system. In this system, a full set of corrugated cardboard tests is performed, i.e., an edge crush test, board torsion/shear tests and four-point bending in both directions. The output from the testing protocol includes the deteriorated properties of cardboard due to crushing, which is an intrinsic side effect that appears in cardboard while converting.

**Figure 3:**
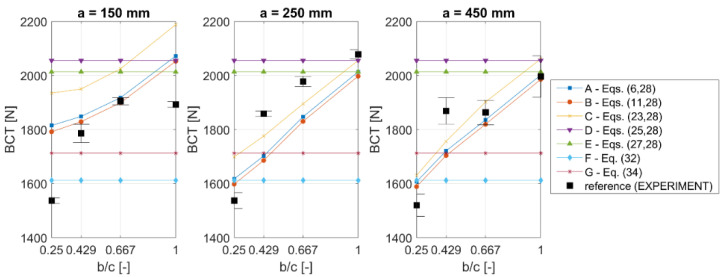
Box compression strength of simple flap boxes with different heights: 150 mm, 250 mm and 450 mm (for cases A–E k = 0.5 and r = 0.75; for case F $\hat{k}$ = 2.028 and r = 0.746; for case G $\overset{ˇ}{k}$ = 5.874 and r = 0.746).

Figure 3 and Table 3 show the mean values of the ultimate load measured at the box failure (BCT) together with ±1σ (one standard deviation) compared with the computed estimation of BCT obtained by various analytical methods (cases A–G, see Table 1).The used values of k and r are the typical literature values, namely k=0.5 and r=0.75. In Table 3, in columns A–G, the error is computed according to the following expression:

(39) $$error=\left|\frac{{\underline{BCT}}_{exp}\;-BCT_{est}\;}{{\underline{BCT}}_{exp}\;}\cdot 100\%\right|$$

where ${\underline{BCT}}_{exp}$ is an experimental average value of the box compression strength for a particular box and $BCT_{est}$ is its counterpart estimated by approaches A–G. Asterix (*) is used to mark the best solution in a row (lowest error); approach B has the largest number of best solutions (four out of 12 box designs). The mean errors obtained for cases A–C are lower than 8%, while for cases D–G the errors are about 11% and more.

The following observations can be made based on the laboratory tests of boxes of different sizes. For boxes with a height of 150 mm, the change in strength was about 19% (1893 N for 250×250 mm vs. 1533 N for 400×100 mm). For boxes with a height of 250 mm, the change increased to 26% (2078 N for 250×250 mm vs. 1537 N for 400×100 mm). The change was 24% for boxes with a height of 450 mm (1996 N for 250×250 mm vs. 1520 N for 400×100 mm).

### 3.2. Reduction of the Estimation Error—Optimal Parameters

**Figure 4:**
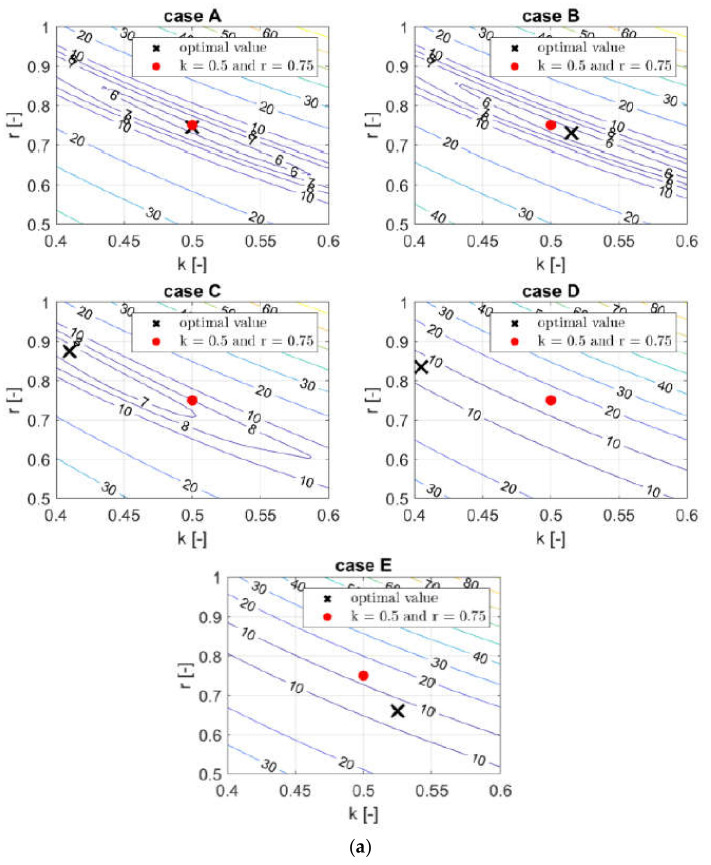

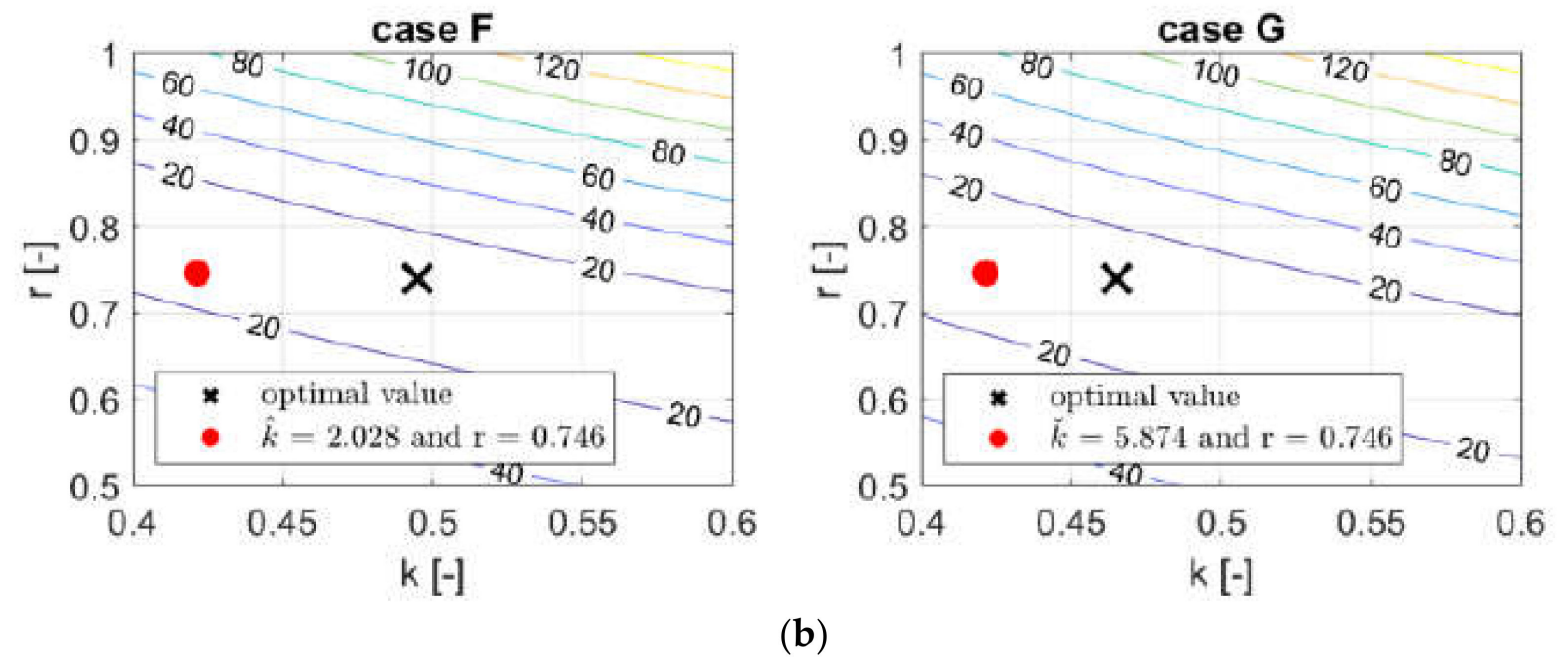
Contour plots of the mean error obtained for cases A–G for: (**a**) k and r (cases A–E) and (**b**) $\hat{k}\left(k,r\right)$ or $\overset{ˇ}{k}\left(k,r\right)$ and r (cases F–G).

In order to verify how much the estimation error depends on the constants (k,r) assumed in the analytical approaches (see Table 1), computations for different sets of constants were performed. For approaches A–E, the values of k and r (see Equation (28)) were modified, and for approaches F–G, the values of r and $\hat{k}\left(k,r\right)$ or $\overset{ˇ}{k}\left(k,r\right)$ (see Equations (32)–(35)) were modified. It should be noted that typical literature values of k and r are 0.50 and 0.75, respectively (see Equation (38)). Consequently, the typical literature values of $\hat{k}$ and r in case F are 2.028 and 0.746, respectively, and in case G, $\overset{ˇ}{k}$ and r are 5.874 and 0.746, respectively. The mean errors obtained by systematic computations are presented by contour plots in Figure 4a for cases A–E and in Figure 4b for cases F–G. The values were computed by averaging the magnitudes from Equation (39) for 12 box designs. The interval of *k* was assumed to be from 0.4 to 0.6, and for *r* it was assumed to be from 0.5 to 1.0.

Table 4 presents the lowest computed errors of cases A–G and the corresponding optimal parameters. The values of k, r,$\;\hat{k}\left(k,r\right)$ and $\overset{ˇ}{k}\left(k,r\right)$ (see Equations (32) and (36)) are optimal solutions obtained by the systematic search in the k−r space (see Figure 4). The lowest errors are obtained by approaches A and B, with errors lower than 6%. Furthermore, it may be observed that the optimal values of constants for approaches D–G reach the limit of 8.04%. Note that in Figure 4 all surfaces have a characteristic valley, in which optimal values could be obtained for different pairs of parameters k and r. In cases A–C, the valley is limited to a smaller area, while for D–F optimal areas exist for the whole range of analyzed parameters. For comparison, Table 5 presents the errors obtained while using typical values of constants, taken from the literature.

**Figure 5:**
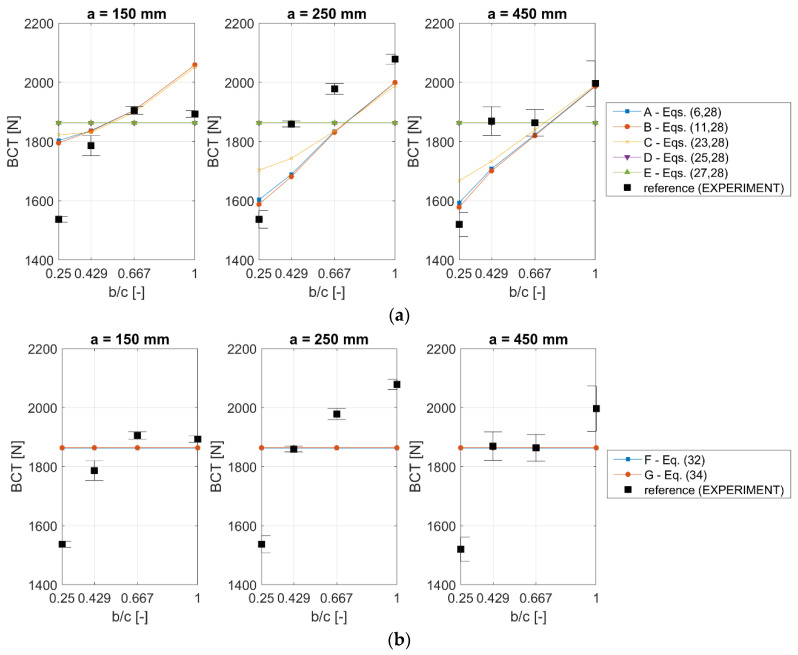
Compressive strength of simple flap boxes with different heights: 150 mm, 250 mm and 450 mm, computed for optimal ratios of k, r, $\hat{k}$ and $\overset{ˇ}{k}$ for: (**a**) cases A–E and (**b**) cases F–G (see Table 4).

Having the typical literature and optimal values of the parameters (k, r, $\hat{k}$ and $\overset{ˇ}{k}$) (see Table 4 and Table 5), the analytical solutions for particular analytical approaches may be revalidated for the considered 12 box designs. The results are presented in Figure 5a for cases A–E and in Figure 5b for cases F–G. As expected, in cases D–G, the same value was obtained for each design, while cases A–C reproduced the experimental trends. Cases A and B are close to each other, while case C differs from them for box designs with low b/c values (0.25 and 0.429).

## 4. Discussion

In this research study, all boxes had the same in-plane circumferences, i.e., 1000 mm (see Table 3 (a, b and c columns)). This feature gives exactly the same value for the BCT estimate according to McKee’s formula (approaches F–G) and in cases with simplified buckling (D–E), where the buckling panel is assumed to be square (see Figure 3 and Figure 5). On the contrary, the test results presented in Figure 2 and Figure 3 (black squares) clearly show that the BCT values obtained experimentally differed across various boxes. These differences are essential, and in selected cases, as described above, they vary from 19% to 26%. In Figure 3, as expected, the results for cases D–G have the same values; those analytical solutions are not sensitive to the b/c change. Those solutions are sensitive both to different in-plane shapes and to the height of the box, if the circumference is constant (Z=const). Notice that cases A–C present a correct result in line with the experimental results, namely, the computational results are sensitive to b/c change, as compared with the black squares in Figure 3.

The correction factors discussed in Section 2 may be optimally selected, or typical (literature) values may be used. As shown in Section 3, their values influence the error, as can be seen in Table 4 and Table 5. While comparing Figure 3 and Figure 5 and inspecting Table 4 and Table 5, it may be noted that the typical constants of k and r for approaches A–B give a very good accuracy (compared to the optimal values). On the other hand, as shown, the typical constants used in the McKee formulas (F–G approaches) may be modified to get a higher accuracy for the boxes made of 3B400 cardboard quality. However, the improvement does not give a lower error than the A–B approach (~5.8% vs. ~8%, see Table 4 and Table 5). Note that, in Figure 5, only approaches A–C tend to follow the trend of the experimental results (black squares) and that in other cases the values of the predicted compressive strength are equal to each other (see D–E in Figure 5a and F–G in Figure 5b).

In this study, the difference between the calculated critical load without (case A) and with transversal shear effects (case B) is not very apparent (see Table 2 and Figure 3 or Figure 5). This is because, in our specific case, the corrugated cardboard (wave B, grammage 400 g/m^2^) is not printed, so the impact of crushing on the material parameters is negligible. In the case of printed, and thus crushed, corrugated cardboard, both the transverse stiffness and the effective thickness are smaller, so the differences are also greater.

Given these results, the conclusion can be drawn that the most detailed buckling approach (see Equation (11)—case B) has the highest accuracy compared to other approaches, including McKee’s long and short formula. Unfortunately, the potential for using detailed buckling in packaging design remains unexplored.

Future studies will be devoted to selected aspects of more advanced approaches to modeling the top-to-bottom strength of the corrugated cardboard boxes considered here, among others a strength decrease of boxes due to an unintentional crushing during material converting.

## 5. Conclusions

This paper presented a detailed analysis of both simplified and advanced methods used for estimating the compressive strength of flap boxes made of corrugated cardboard. The purpose of the work was to evaluate the applicability of these methods and to determine the value of the estimation error resulting from the use of various simplifications. This study focused on the description of the critical buckling load, which is one of the two main components determining the load capacity of corrugated cardboard packaging. The paper also compared the McKee estimation method, proposed in the 60s, with modern ways of predicting the compressive strength of boxes (see Table 3).

The results showed that, for certain packaging dimension proportions, the simplified description of the critical load used in the selected formulas gave estimation errors at a level of 8–15%, as seen in Table 5. On the contrary, the estimation errors for the most precise approach, when orthotropy, aspect ratio and transverse shearing stiffness were included, gave a mean estimation error of about 6%, as seen in Table 4 and Table 5. The use of more precise formulas requires more laboratory tests on corrugated cardboard but in return provides more accurate results than the McKee-type formulas do.

## Figures and Tables

**Table 1:** Analytical approaches for the determination of the box compressive strength.

Case	Box Compressive Strength [N]	Buckling Force	a≠b	Orthotropy	Transverse Shear
A	$BCT=2kECT^{r}\left[\gamma_{b}{\left(P_{cr}^{b}\right)}^{1-r}b+\gamma_{c}{\left(P_{cr}^{c}\right)}^{1-r}c\right]$	Equation (6)	+	+	−
B		Equation (11)	+	+	+
C		Equation (23)	+	±	+
D		Equation (25)	−	−	−
E		Equation (27)	−	±	−
F	$BCT=\hat{k}\;ECT^{r}{\left(\sqrt{D_{11}D_{22}}\right)}^{1-r}Z^{2r-1}$	−	−	±	−
G	$BCT=\overset{ˇ}{k}\;ECT\;h^{2\left(r-1\right)}Z^{2r-1}$	−	−	−	−

**Table 2:** Mechanical properties (stiffnesses, thickness and ECT value) of *3B400* cardboard quality.

$D_{11}\;[\mathrm{Nmm}]$	$D_{22}\;[\mathrm{Nmm}]$	$D_{12}\;[\mathrm{Nmm}]$	$D_{66}\;[\mathrm{Nmm}]$	$A_{44}\;[N/\mathrm{mm}]$	$A_{55}\;[N/\mathrm{mm}]$	h [mm]	ECT [N/mm]
3269.0	1785.0	717.5	602.2	16.81	54.77	2.85	5.72

$D_{11}$, $D_{22}$, $D_{12}$, and $D_{22}$—flexural stiffnesses, see Equations (8)–(9), $A_{44}$ and $A_{55}$—transverse shear stiffnesses, see Equation (14), *h*—cardboard thickness and ECT—measured value from edge crush test.

**Table 3:** Experimental data (dimensions and box compression strength) and computational errors according to different analytical approaches (see Table 1) for 3B400 cardboard quality; for cases A–E k = 0.5 and r = 0.75; for case F $\hat{k}$ = 2.028 and r = 0.746; for case G $\overset{ˇ}{k}$ = 5.874 and r = 0.746.

No.	b [mm]	c [mm]	a [mm]	BCT ± σ [N]	A [%]	B [%]	C [%]	D [%]	E [%]	F [%]	G [%]
1.	250	250	150	1893 ± 10.9	9.5	8.4	15.6	8.6	6.4 *	14.8	9.5
2.	200	300	150	1905 ± 13.2	0.7	0.3 *	6.3	7.9	5.7	15.4	10.1
3.	150	350	150	1786 ± 33.7	3.5	2.4 *	9.2	15.1	12.7	9.7	4.1
4.	100	400	150	1533 ± 9.80	18.1	16.6	25.9	33.7	31.0	4.9 *	11.4
5.	250	250	250	2078 ± 17.2	3.1	3.9	1.1 *	1.1 *	3.1	22.4	17.6
6.	200	300	250	1978 ± 18.7	6.6	7.5	4.2	3.9	1.8 *	18.5	13.4
7.	150	350	250	1859 ± 9.90	8.4	9.3	4.5 *	10.6	8.3	13.3	7.9
8.	100	400	250	1537 ± 29.5	5.2	4.0 *	10.5	33.7	31.0	4.9	11.4
9.	250	250	450	1996 ± 76.7	0.3 *	0.5	3.3	3.0	0.9	19.2	14.2
10.	200	300	450	1863 ± 45.3	1.5 *	2.3	2.1	10.3	8.1	13.5	8.1
11.	150	350	450	1869 ± 48.6	7.9	8.8	6.0 *	10.0	7.7	13.7	8.4
12.	100	400	450	1520 ± 41.0	5.7	4.5 *	7.3	35.2	32.5	6.1	12.7
				mean [%]:	5.87	5.71 *	7.99	14.4	12.4	13.0	10.7

* Denotes the lowest value in the row. a, b, and c—box dimensions, i.e., height, width, and length, respectively, BCT ± σ—measured values from box compression test with standard deviation, and A–G—see Table 1.

**Table 4:** Mean errors for cases A–G; values of k and r obtained from the optimization process.

Case	Optimal Parameters		Mean Error [%]
	[-]	[-]	
A	k=0.500	r=0.745	5.79
B	k=0.515	r=0.730	5.67
C	k=0.410	r=0.875	6.41
D	k=0.405	r=0.835	8.04
E	k=0.525	r=0.660	8.04
F	$\;\hat{k}=2.455\;\left(k=0.495\right)$	r=0.740	8.04
G	$\overset{ˇ}{k}=6.857\;\left(k=0.465\right)$	r=0.740	8.04

**Table 5:** Mean errors for cases A–G; values of k and r taken from literature.

Case	Constants from the Literature		Mean Error [%]
	[-]	[-]	
A	k=0.50	r=0.75	5.87
B			5.71
C			7.99
D			14.4
E			12.4
F	$\;\hat{k}=2.028\;\left(k=0.422\right)$	r=0.746	13.0
G	$\overset{ˇ}{k}=5.874\;\left(k=0.422\right)$	r=0.746	10.7
